# Self-defect-passivation by Br-enrichment in FA-doped Cs_1−*x*_FA_*x*_PbBr_3_ quantum dots: towards high-performance quantum dot light-emitting diodes

**DOI:** 10.1038/s41598-020-71666-8

**Published:** 2020-09-08

**Authors:** Young Ran Park, Sangwon Eom, Hong Hee Kim, Won Kook Choi, Youngjong Kang

**Affiliations:** 1 Institute of Nano Science and Technology (INST), Hanyang University, Seongdong-gu, Seoul, 04763 South Korea; 2 Department of Chemistry, Hanyang University, Seongdong-gu, Seoul, 04763 South Korea; 3 Research Institute for Natural Sciences, Hanyang University, Seoul, 04763 South Korea; 4 Center for Opto-Electronic Materials and Devices, Korea Institute of Science and Technology (KIST), Seongbuk-gu, Seoul, 02792 South Korea; 5 Department of Materials Science and Engineering, Yonsei University, Seodaemun-gu, Seoul, 03722 South Korea

**Keywords:** Materials for optics, Nanoscale materials

## Abstract

Halide vacancy defect is one of the major origins of non-radiative recombination in the lead halide perovskite light emitting devices (LEDs). Hence the defect passivation is highly demanded for the high-performance perovskite LEDs. Here, we demonstrated that FA doping led to the enrichment of Br in Cs_1−*x*_FA_*x*_PbBr_3_ QDs. Due to the defect passivation by the enriched Br, the trap density in Cs_1−*x*_FA_*x*_PbBr_3_ significantly decreased after FA doping, and which improved the optical properties of Cs_1−*x*_FA_*x*_PbBr_3_ QDs and their QD-LEDs. PLQY of Cs_1–*x*_FA_*x*_PbBr_3_ QDs increased from 76.8% (*x* = 0) to 85.1% (*x* = 0.04), and L_*max*_ and CE_*max*_ of Cs_1–*x*_FA_*x*_PbBr_3_ QD-LEDs were improved from L_*max*_ = 2880 cd m^−2^ and CE_*max*_ = 1.98 cd A^−1^ (*x* = 0) to L_*max*_ = 5200 cd m^−2^ and CE_*max*_ = 3.87 cd A^−1^ (*x* = 0.04). Cs_1–*x*_FA_*x*_PbBr_3_ QD-LED device structure was optimized by using PVK as a HTL and ZnO modified with b-PEI as an ETL. The energy band diagram of Cs_1–*x*_FA_*x*_PbBr_3_ QD-LEDs deduced by UPS analyses.

## Introduction

Colloidal lead halide perovskites have recently emerged as promising materials for light-emitting diodes (LEDs), because of their unique advantages of a tunable emission wavelength, high color purity, and low temperature and cost-effective solution process capability^1–9^. These perovskites exhibited tunable emission wavelength (400 nm ≤ λ ≤ 780 nm) due to their compositional versatility, and high photoluminescence quantum yields (PLQYs) across the entire visible range with narrow emission bandwidth (FWHM < 20 nm)^1,10,11^. While the first perovskite-based LEDs were reported in 1994^12^, their significant progress has been made in recent years using lead halide perovskite quantum dots (QDs)^5,10,13,14^. During the past 3 years, the external quantum efficiency (EQE) of perovskite LEDs significantly increased from 0.01–0.1% up to 21.6%^5,13–18^. MAPbBr_3_ LEDs have shown high electroluminescent (EL) performance comparable to that of organic LEDs^10^. MAPbBr_3_ perovskites, however, are susceptible to heat and moisture^19–21^. As a solution for these problems, MA cations have been replaced with the larger cations, FA (formamidinium) or Cs^22^. For example, incorporation of FA increased efficiency and stability of infrared LEDs (EQE_*max*_ = 11.7%^18^ and 21.6%^15^) and green LEDs (current efficiency, CE_*max*_ = 17.1 cd A^−1^)^16^. Additionally, the mixed cations of FA and Cs and their stoichiometric control have been investigated to improve device stability and efficiency^23–25^. FA-Cs mixed-cation perovskite LEDs have shown a CE_*max*_ of 10.09–14.5 cd A^−1^^23,26^. Similarly, a small amount of FA cation was incorporated as a dopant to further improve the PL of CsPbBr_3_ QDs^6^.

The trap state control is important for improving the efficiency of perovskite LED devices. Due to labile halide migration, the trap states generated in lead halide perovskites are generally believed to be associated with halide vacancies^27^. Hydrogen bonding can be used to stabilize halide ions in perovskites^15,21,28–30^. In this case, the hydrogen bonding strength should be carefully tweaked. Xu et al*.* showed that the amino-functionalized passivation agents with relatively weak hydrogen bonding ability were more preferential to interact with defects than organic cations, so that they much improved the defect passivation efficiency^15^. Similarly, the internal hydrogen bonding between halides and ammonium cations (MA or FA) within the perovskite framework affects the geometry of perovskites^11^.

Herein, we report the effects of the FA doping on CsPbBr_3_ QD-LEDs. We found that partial substitution of Cs with FA led to the significant increase of Br concentration in Cs_1−*x*_FA_*x*_PbBr_3_ framework by hydrogen bonding and ionic interaction between FA and Br^–^, and which dramatically decreased the defects in Cs_1−*x*_FA_*x*_PbBr_3_. Accordingly, inverted-type perovskite LEDs prepared with Cs_1−*x*_FA_*x*_PbBr_3_ QD in ambient condition exhibited much better performance than those prepared with undoped CsPbBr_3_ QDs. The performance of QD-LEDs with Cs_1−*x*_FA_*x*_PbBr_3_ at the optimized composition (*x* = 0.04) exhibited the maximum luminance (L_*max*_) of 5200 cd m^−2^ at 5.3 V and the maximum current efficiency (CE_*max*_) of 3.87 cd A^−1^ at 5.0 V. These are much better than those values for undoped (*x* = 0) and over-doped (*x* = 0.055) ones: L_*max*_ = 2880 cd m^−2^ at 6.2 V and CE_*max*_ = 1.98 cd A^−1^ at 5.9 V for CsPbBr_3_, and L_*max*_ = 2250 cd m^-2^ at 5.6 V and CE_*max*_ = 2.73 cd A^−1^ at 5.0 V Cs_0.945_FA_0.055_PbBr_3_, respectively. This FA doping strategy enables us not only to suppress the non-radiative recombination in luminance layer to improve the performance of QD-LEDs but also to realize the high efficiency in optoelectronic devices.

## Results and discussion

**Figure 1:**
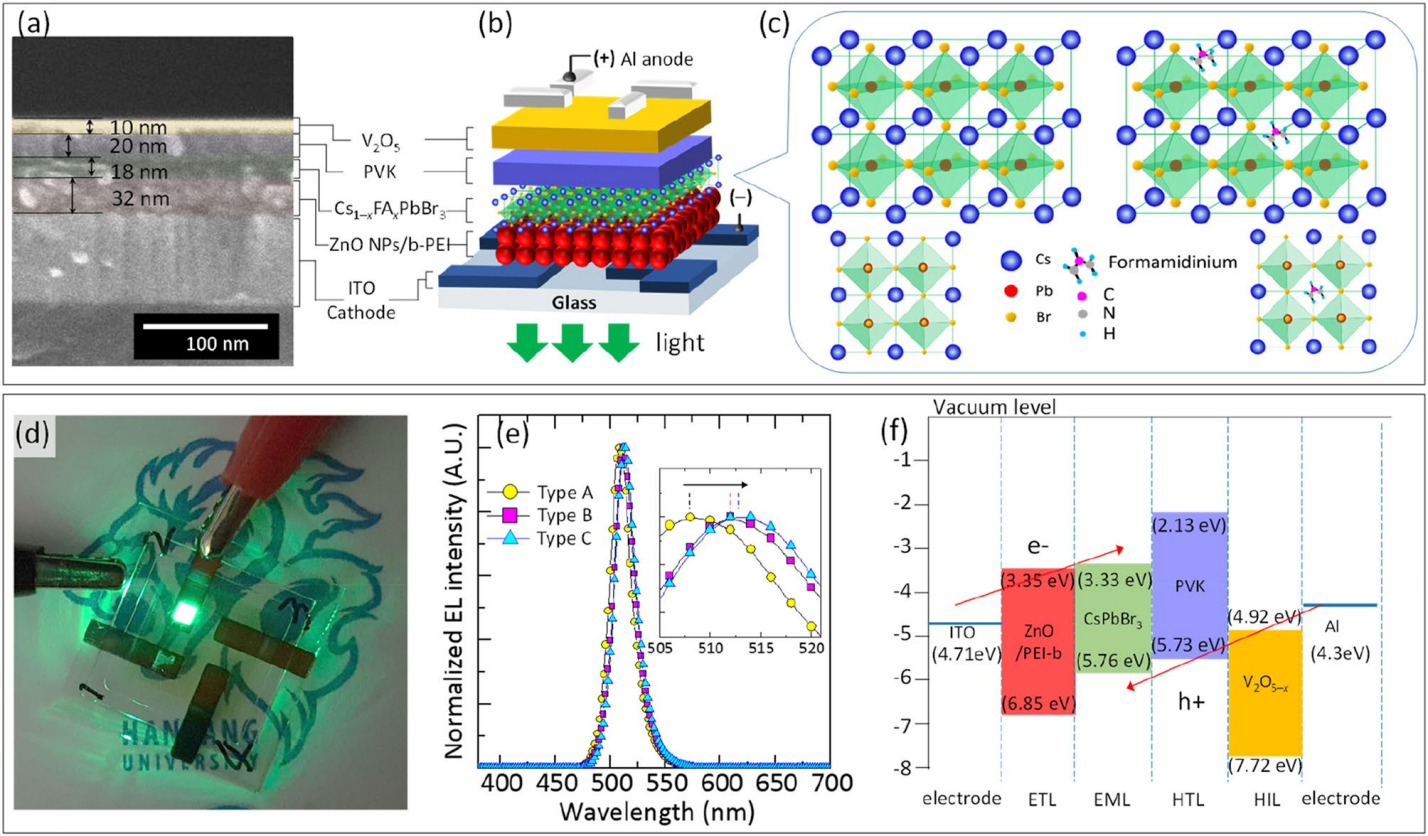
Solution-processed inverted-type Cs_1−*x*_FA_*x*_PbBr_3_ QD-LEDs. (**a**) Cross-section SEM image of a QD-LED, (**b**) Schematic device structure of the QD-LED, (**c**) Crystal structures of undoped and FA doped CsPbBr_3_. (**d**) Luminance photograph of a Cs_1−*x*_FA_*x*_PbBr_3_ QD-LED with an active area of 2 × 2 mm^2^ (at 4.1 V). (**e**) Maximum EL spectra of Type A, Type B, and Type C QD-LEDs working at 5.9 V, 5.0 V, and 5.0 V, respectively. (**f**) Schematic energy level diagram of the layers used for the QD-LEDs.

Figure 1a–b show the stack configuration of a solution-processed inverted-type QD-LED with multilayer heterojunctions of ZnO NCs modified with b-PEI (ZnO/b-PEI), Cs_1−*x*_FA_*x*_PbBr_3_ perovskite QDs, PVK, and V_2_O_5−*x*_, which were sequentially spin-coated on ITO-coated glass substrates under the ambient condition, for electron transport layer (ETL), luminescence layer, hole transport layer (HTL), and hole injection layer (HIL), respectively. The thicknesses of ZnO/b-PEI, Cs_1−*x*_FA_*x*_PbBr_3_, PVK, and V_2_O_5−*x*_ layers was approximately 32, 18, 20, and 10 nm, respectively, which were determined by the cross-sectional SEM image shown in Fig. 1a. Figure 1c presents a schematic illustration of Cs_1−*x*_FA_*x*_PbBr_3_ with a cubic structure consisting of the Pb cation in sixfold coordination surrounded by an octahedron of Br anions and the Cs cation (and partial substitution of FA) in 12-fold cuboctahedral coordination. Figure 1d shows the photograph of the Cs_1−*x*_FA_*x*_PbBr_3_ QD-LED device fabricated on a 2 × 2 mm^2^ active area exhibiting uniform emission. Figure 1e presents the normalized electroluminescence (EL) spectra of devices prepared using Cs_1−*x*_FA_*x*_PbBr_3_ QDs. The EL peaks located at 508, 512, and 513 nm for Type A (*x* = 0), Type B (*x* = 0.04) and Type c (*x* = 0.055), respectively. All EL spectra show narrow emission (FWHM = 19 nm) and high color purity, which is solely attributed to the band-edge emission of Cs_1−*x*_FA_*x*_PbBr_3_ QDs with a slightly redshifted emission from the PL spectrum taken in the QD colloidal solution^32^. Additionally, no notable parasitic emission originated from the PVK layer was observed (Fig. 1e)^31^. Figure 1f shows the electronic energy level diagram (with respect to the vacuum level) of the layers applied for the QD-LEDs. The ionization energy (*IE*) and the electron affinity (*EA*) of these layers were estimated from the UV–visible absorption spectra (not shown here) and UPS analysis, which were in good agreement with the previous studies^3,31,33–35^.

**Figure 2:**
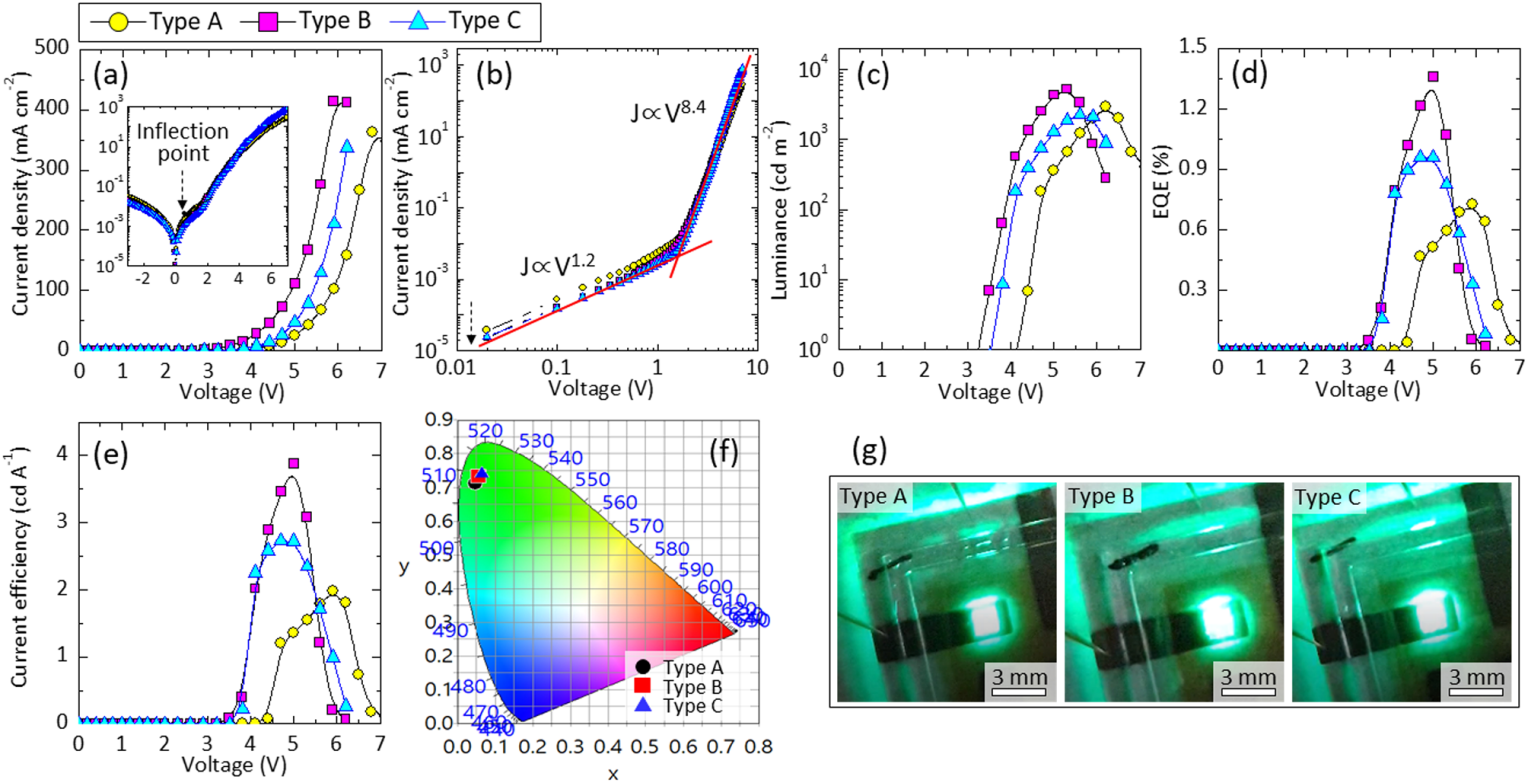
Electrical and electroluminescent characteristics of the QD-LEDs of Type A, Type B, and Type C. (**a**) *J*–*V* characteristic curves. The inset is *J*–*V* curves of semi-logarithmic axes. (**b**) *J*–*V* characteristic curves plotted on double-logarithmic axes. (**c**) Luminance, (**d**) external quantum efficiency, and (**e**) current efficiency plotted as a function of applied bias voltage. (**f**) Commission International de l’Éclairage 1931 (CIE) coordinates of EL emissions obtained from QD-LEDs, measured at operating voltages of 5.6 V (Type A), 4.4 V (Type B), and 5.1 V (Type C), respectively. (**g**) Photographs showing bright and uniform EL emission at the operating voltage of 5.6 V (Type A), 4.4 V (Type B), and 5.1 V (Type C), respectively.

The electrical and electroluminescent performances of Cs_1−*x*_FA_*x*_PbBr_3_ QD-LEDs were examined by measuring the *J*–*V*–*L* characteristic curves. Figure 2a shows the *J*–*V* characteristic curves of Cs_1−*x*_FA_*x*_PbBr_3_ QD-LEDs. All QD-LEDs exhibited high electrical rectification behavior with an inflection point around at 0.54 V and steeper increase of the current density above the inflection point (inset of Fig. 2a). In Fig. 2b, *J*–*V* characteristic curves plotted on double-logarithmic axes. In the Ohmic conduction region (*J* $\propto$*V*^1.2^), the leakage current of devices doped with FA (Type B and Type C) was slightly lower than that of Type A. In the trap-limited conduction region, the *J*–*V* curve slops for all QD-LEDs (*J* $\propto$*V*^8.4^) were slightly deviated from the typical power law (*J* $\propto$*V*^7^), which was attributed to similar charge injection/transport energy band diagram of the QD-LEDs structure. Interestingly, the Type B and C QD-LEDs exhibited higher luminance, higher current efficiency (CE), higher external quantum efficiency (EQE), and lower turn-on voltage than those of Type A, as shown in Fig. 2c–e respectively. The turn-on voltage (calculated with a luminance of 1 cd m^−2^) was 4.1, 3.2, and 3.5 V for Type A, B and C, respectively. Originally, the maximum luminance (L_*max*_) of the Type A was 2880 cd m^−2^ at 6.2 V. It significantly increased to 5200 cd m^−2^ (at 5.3 V) when CsPbBr_3_ was slightly doped with FA (*x* = 0.04, Type B). We attribute this enhancement to the decreased trap density by doping with FA. However, L_*max*_ decreased to 2250 cd m^−2^ at 5.6 V when CSPbBr_3_ was over-doped with FA (*x* = 0.055, Type C). This is because the valence band maximum (VBM) of the CsPbBr_3_ is slightly upshifted by FA doping, which makes difficult inject electrons (this will be discussed in Fig. 6). The EQE and CE were also maximized for Type B devices (Fig. 2d,e). The highest EQE of Type B device was EQE_*max*_ = 1.36%, which was much higher than that of Type A (EQE_*max*_ = 0.72%) and Type C (EQE_*max*_ = 0.96%), respectively (Fig. 2d). At the same time, Type B device showed much better CE_*max*_ = 3.87 cd A^−1^ than that of Type A (CE_*max*_ = 1.98 cd A^−1^) and Type C (CE_*max*_ = 2.73 cd A^−1^) (Fig. 2e). All devices exhibited a bright and uniform pure green color from the entire pixel area under bias voltage of 4.0–6.0 V, and their corresponding CIE (Commission Internationale de l’Eclairage 1931) chromaticity coordinates were Type A (0.048, 0.711), Type B (0.057, 0.733), and Type C (0.064, 0.742), respectively (Fig. 2f,g).

**Figure 3:**
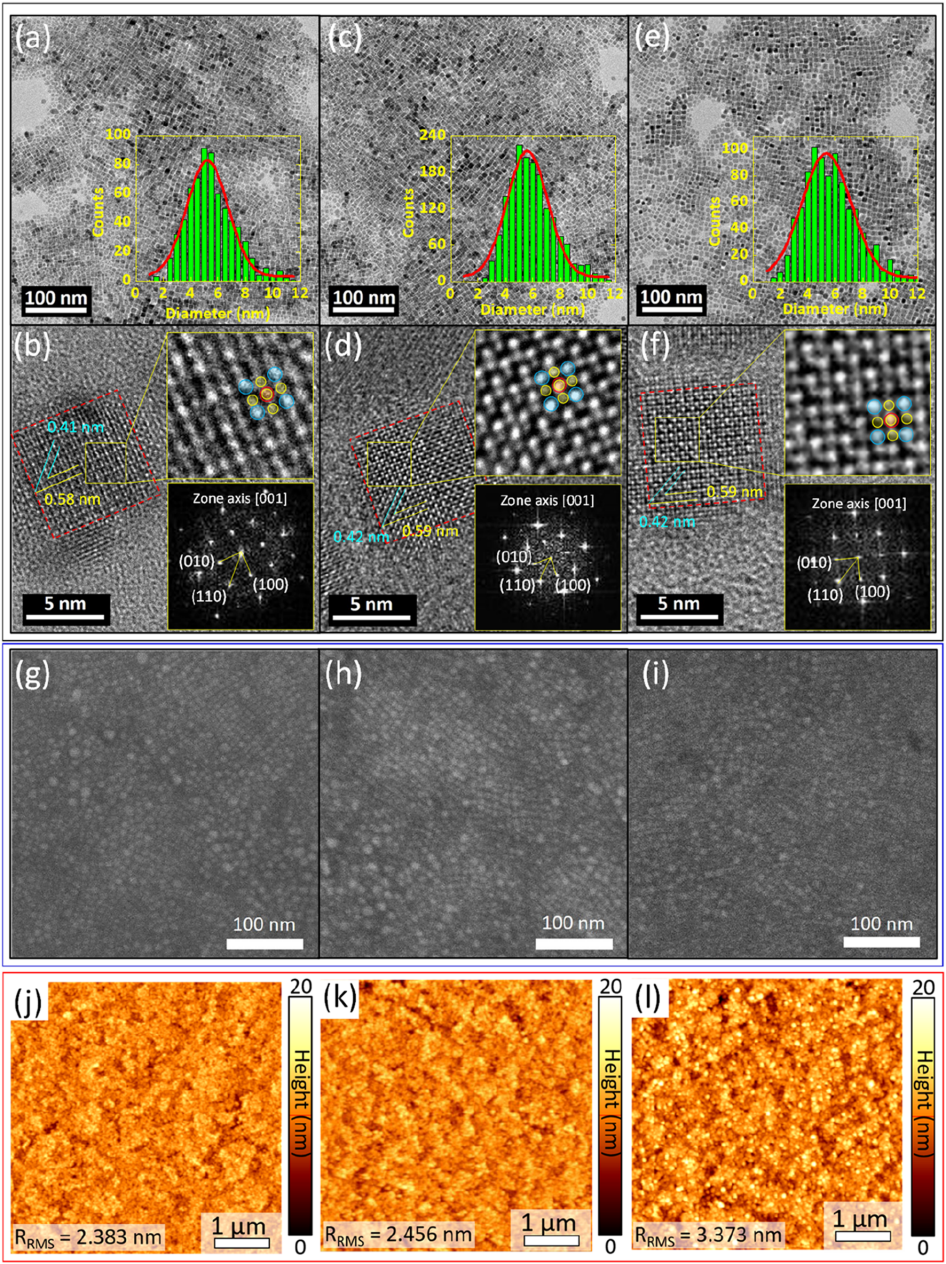
Cs_1−*x*_FA_*x*_PbBr_3_ QDs: (**a**,**b**) Type A, (**c**,**d**) Type B, and (**e**,**f**) Type C. (**a**,**c**, and **e**) TEM micrographs of the representative Cs_1−*x*_FA_*x*_PbBr_3_ QDs. Insets show the size distribution of QDs. (**b**,**d**, and **f**) HR-TEM micrographs showing the lattice spacing of corresponding Type A, B and C Cs_1−*x*_FA_*x*_PbBr_3_ QDs. Insets are HR-TEM micrographs of Cs_1−*x*_FA_*x*_PbBr_3_ QDs along the [001] zone axis (top) and Fast-Fourier Transform (FET) patterns (bottom) indicating the cubic structure of Cs_1−*x*_FA_*x*_PbBr_3_ (blue, red, and yellow balls represent Cs, Pb, and Br ions, respectively). (**g**–**l**) Surface analyses of Cs_1−*x*_FA_*x*_PbBr_3_ QD films on b-PEI/ZnO/ITO obtained by (**g**–**i**) FE-SEM and (**j**–**l**) AFM (5 × 5 μm^2^): (**g**,**j**) Type A, (**h**,**k**) Type B, and (**i**,**l**) Type C.

Above all data clearly suggest that FA doping significantly improves the device performances including luminescence, EQE and CE of Cs_1–*x*_FA_*x*_PbBr_3_ QD solutions. To understand the effects of FA doping on CsPbBr_3_ QDs, we first investigated them with TEM. Figure 3a,c, and e show representative TEM micrographs of Cs_1–*x*_FA_*x*_PbBr_3_ QDs. They formed the well-defined cubic particles with an average diameter of *d*_avg_ = 6.0 ± 0.16 nm, 6.5 ± 0.19 nm, and 6.5 ± 0.21 nm for Type A, B and C, respectively. HR-TEM images clearly show the cubic crystal structure of Type A QDs (Fig. 3b). The measured *d*-spacing for (100) and (110) plane was 0.58 nm and 0.41 nm, respectively. With FA doping, the *d*-spacing for (100) and (110) plane slightly increased to 0.59 nm and 0.42 nm for both Type B and Type C (Fig. 3d,f). The enlarged HR-TEM images in the top-insets of Fig. 3b,d, and f well match with the atomic arrangement of cubic Cs_1–*x*_FA_*x*_PbBr_3_ QDs crystal. The bottom-insets of Fig. 3b,d, and f show the Fast Fourier Transform (FFT) patterns confirming the cubic crystal structure of Cs_1–*x*_FA_*x*_PbBr_3_ QDs. These data also well matched with X-ray diffraction (XRD) patterns presented in Supplementary Figure S1. As shown in Supplementary Figure S1, the peaks with 2θ values of 15.1, 21.5, 26.4, 30.4, 34.2, 37.6, 43.8, 46.5 correspond to the (100), (110), (200), (210), (211), (220), and (300) planes of the CsPbBr_3_ crystal, respectively. All these data suggest that FA doping didn’t affect much the crystal structure of Cs_1–*x*_FA_*x*_PbBr_3_ QDs while *d*-spacing slightly increased, which was attributed to the partial substitution of Cs with FA.

Figure 3g–l show the surface morphology of the Cs_1–*x*_FA_*x*_PbBr_3_ films spin-casted on the b-PEI/ZnO/ITO underlayers. Both SEM and AFM images show uniform and closely packed QDs layer. The averaged rms-roughness was 2.383 ± 0.1 nm (Type A), 2.456 ± 0.1 nm (Type B), and 3.33 ± 0.1 nm (Type C). Supplementary Figure S2 shows FE-SEM images of the surface morphologies of (a) ZnO on ITO, (b) b-PEI on ZnO/ITO, (c) CsPbBr_3_ on b-PEI/ZnO/ITO, (d) PVK on CsPbBr_3_/b-PEI/ZnO/ITO, and (e) V_2_O_5–*x*_ on PVK/CsPbBr_3_/b-PEI/ZnO/ITO. Each step formed smooth and uniform surface with high coverage.

**Figure 4:**
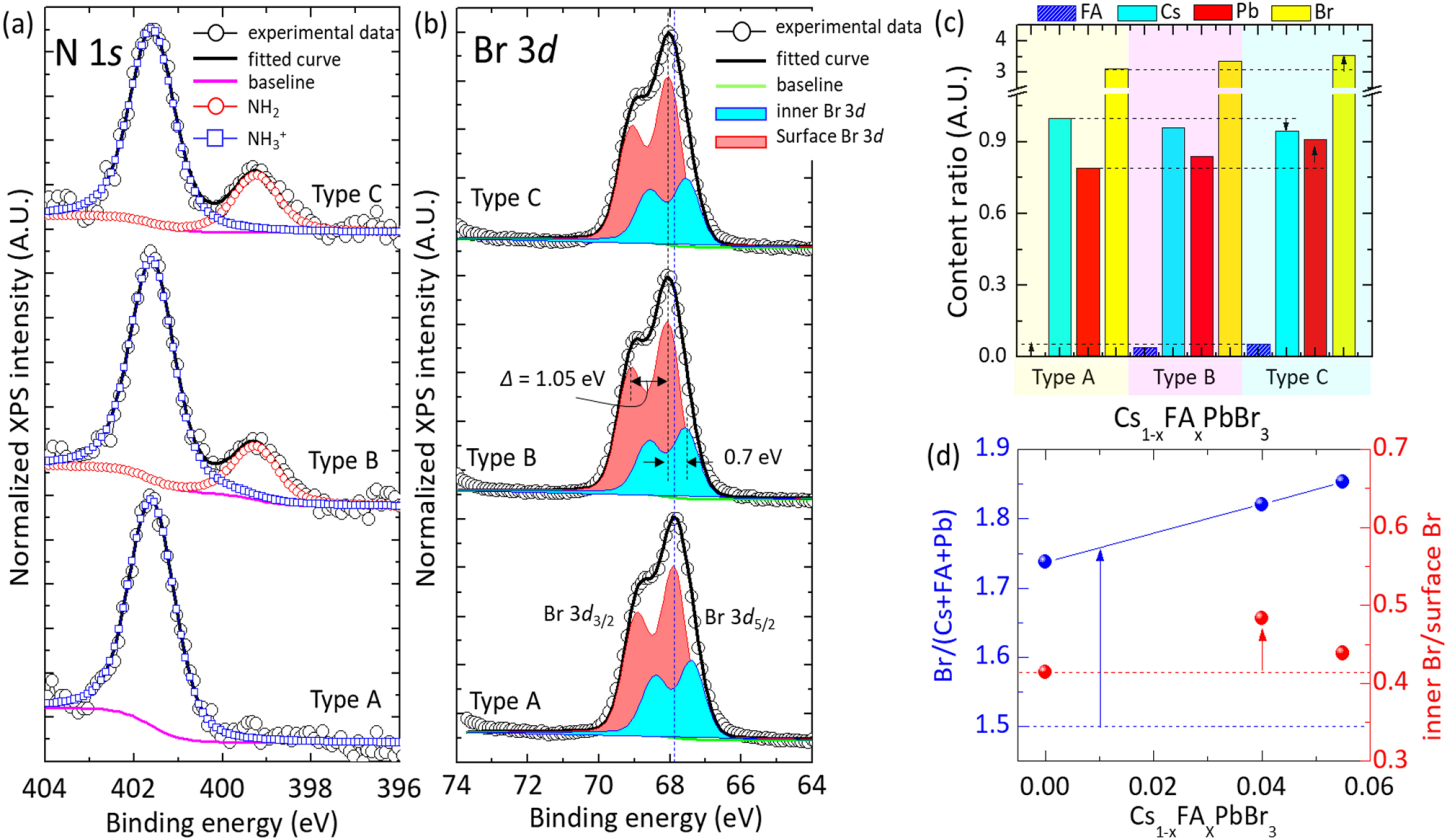
Peak-fitted XPS spectra of Cs_1–*x*_FA_*x*_PbBr_3_ QDs showing (**a**) N 1*s* core level and (**b**) Br 3*d* core level. (**c**) Relative ratio of elements including Cs, FA, Pb, and Br. (**d**) The changes of the anion to cation ratio [Br/(Cs + FA + Pb)] and the inner Br to surface Br ratio [(inner Br)/(surface Br)] in Cs_1–*x*_FA_*x*_PbBr_3_ QDs as a function of FA doping content.

The elemental composition of Cs_1–*x*_FA_*x*_PbBr_3_ QDs was investigated by using XPS. As shown in Supplementary Figure S3a, six major XPS peaks were assigned as Br 3*d*, Pb 4*f*, C 1*s*, N 1*s*, O 1*s*, and Cs 3*d*, respectively. The chemical state of nitrogen was carefully characterized by multiple-peak fitting of the N 1*s* peak using symmetric Voigt functions. Figure 4a shows the characteristic N 1*s* spectra of Cs_1–*x*_FA_*x*_PbBr_3_ QDs (Type A–C). Only ammonium (–NH^3+^) peak was observed at 401.6 eV for Type A, and which is originated from DDAB added as a ligand for synthesis^4,36^. It is notable that the TOAB does not act as a ligand owing to the large steric effect^6^. For Type B and C, however, the primary amine group (–NH_2_) as well as ammonium group (–NH^3+^) was observed at 399.0 eV. The presence of the primary amine group suggests the formation of FA doped Cs_1–*x*_FA_*x*_PbBr_3_ QDs. Based on the XPS analysis, the relative concentration of FA to Cs was determined as 0.04 and 0.055 for Type B and C, respectively. Similarly, XPS data for C 1s also well support the formation of FA doped Cs_1–*x*_FA_*x*_PbBr_3_ QDs (Figure S3b, Supplementary Information). The characteristic peak of FA, C=N (287.6 eV) was observed for Type B and C along with C–C (284.8 eV) and C–N (285.6 eV) peaks, while only two peaks of C–C and C–N bonding were observed for Type A.

The elemental composition including Cs, Pb and Br in Cs_1–*x*_FA_*x*_PbBr_3_ was further investigated (Fig. 4 and Supplementary Figures S3–S5). As summarized in Fig. 4c, it is notable that the concentration of Br was significantly increased by FA doping. Originally, undoped CsPbBr_3_ QDs (Type A) have a composition ratio of Cs:Pb:Br = 1.00:0.79:3.11. The composition ratio for CsPbBr_3_ NC with large size (*d*_avg_ = 8.0 ± 0.16 nm) was Cs:Pb:Br = 1.00:0.73:2.90. In this case, the terminology of CsPbBr_3_ NC was used to clarify that its average size (*d*_avg_ = 8.0 ± 0.16 nm) is larger than the Bohr diameter (*d*_Bohr_ = 7 nm)^7,8,37,38^, while the size of CsPbBr_3_ QD is smaller than the Bohr diameter. More careful analysis of XPS peaks for Br 3*d* revealed that Br^‒^ ions are concentrated at the surface of QD. The concentration of Br^‒^ ions located at the surface (highlighted in red shadow, 3*d*_5/2_ = 67.90 eV and 3*d*_3/2_ = 68.95 eV) was higher than that of the Br^‒^ ions located inside (highlighted in cyan shadow, 3*d*_5/2_ = 67.40 eV and 3*d*_3/2_ = 68.40 eV) (Fig. 4b)^39^. The Br-rich surface of CsPbBr_3_ QD was attributed to the ionic interaction of Br^‒^ ions with ammonium groups in DDAB^6^. The composition ratio of (Cs + FA):Pb:Br was changed to 1.00:0.84:3.35 and 1.00:0.91:3.54 as increasing FA doping concentration to x = 0.04 (Type B) and x = 0.055 (Type C), respectively. In this case, the ratio of anion to cation [Br/(Cs + FA + Pb)] increased almost linearly with the FA content in Cs_1–*x*_FA_*x*_PbBr_3_ (Fig. 4d, the left axis). At the same time, FA doping increased the inner Br content as well as the surface Br content (Fig. 4d, the right axis). The inner/surface Br ratio was maximized for Type B. These results strongly suggest that hydrogen bonding and ionic interaction of FA with Br^‒^ ions led to the increase of Br content. Since FA molecules have both a primary amine group and an iminium group, the hydrogen bonding as well as ionic interaction in Cs_1–*x*_FA_*x*_PbBr_3_ QDs is highly effective^40^.

The effects of Br-enrichment on the optical properties of Cs_1–*x*_FA_*x*_PbBr_3_ QDs have been investigated (Figures S6–S8 and Table S1, Supplementary Information). Supplementary Figure S6 shows the absorbance and PL of Cs_1–*x*_FA_*x*_PbBr_3_ QD solutions. As increasing FA contents, both absorbance and PL were slightly red-shifted. PLQY of Cs_1–*x*_FA_*x*_PbBr_3_ QD solutions varied from 76.8% (Type A) to 85.1% (Type B), and 82.6% (Type C) (Figure S7, Supplementary Information). CsPbBr_3_ NC solution with large size showed a lower PLQY of 67% (Figure S7, Supplementary Information). PLQY data well agreed with TR-PL measurements (Figure S8 and Table S1, Supplementary Information). The PL decay curves were well-fitted with the biexponential decay function consisting of a fast-decay lifetime (*τ*_1_) and a slow-decay lifetime (*τ*_2_) (Table S1, Supplementary Information)^10^. In this case, *τ*_1_ is originated from the initially populated core-state recombination, and *τ*_2_ is related with the surface emission^41^. As summarized in Table S1, *τ*_2_ was dramatically increased by FA doping while *τ*_1_ showed relatively small changes. Hence, the average PL decay times <*τ*> increased from 16.5 ns (Type A) to 30.51 ns (Type B) and 28.51 ns (Type C). Furthermore, the contribution ratio of the surface-related emission (*W*_2_) to the intrinsic core-state recombination emission (*W*_1_) was maximized for Type B. These results strongly suggest that FA doping reduced the trap-states in the Cs_1–*x*_FA_*x*_PbBr_3_ QD, and which was apparently originated from Br-enrichment by FA doping.

**Figure 5:**
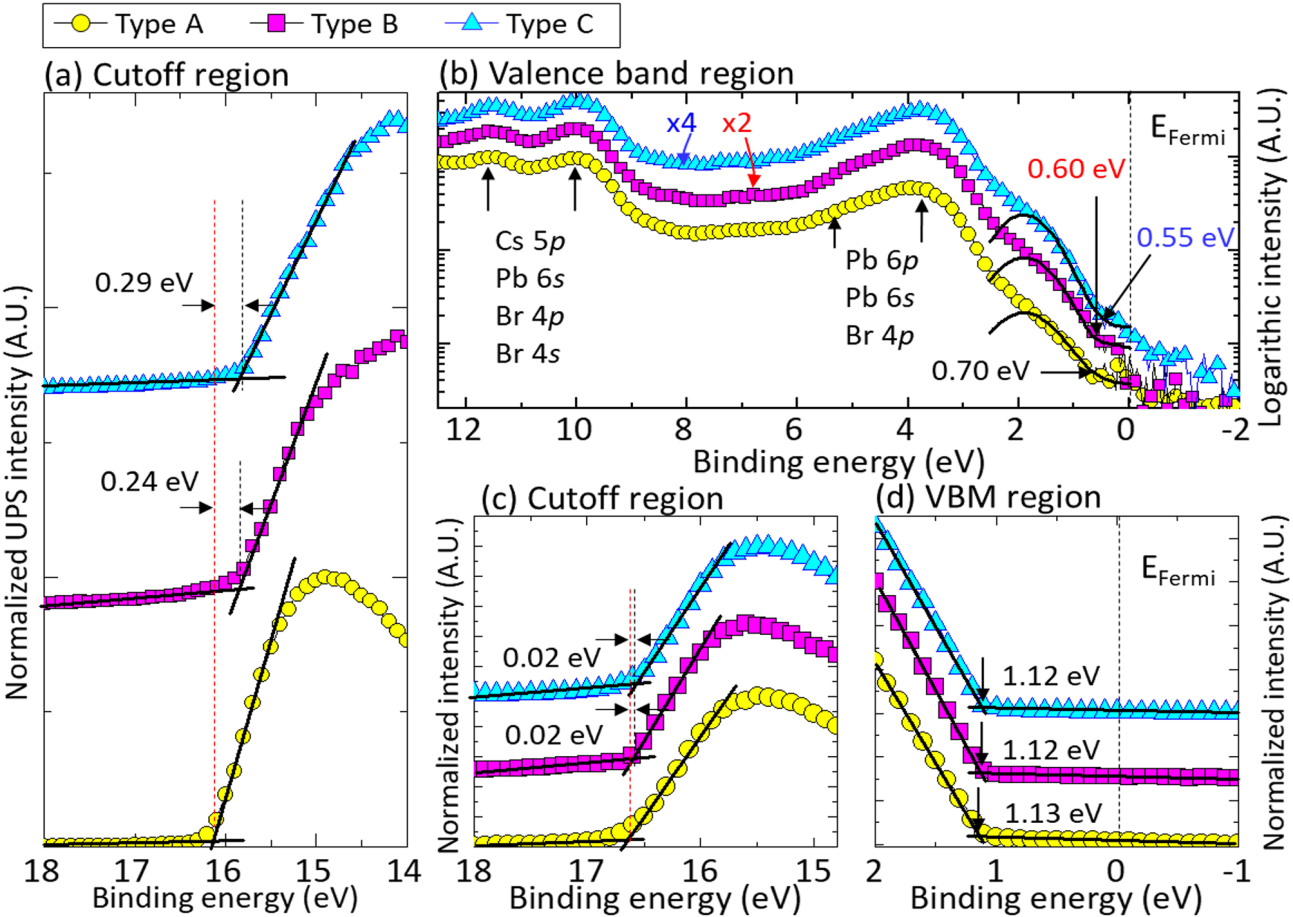
UPS data of multilayer heterojunctions recorded (**a**,**c**) in the low kinetic energy region (the secondary electron cutoff), and (**b**,**d**) in the low-binding-energy region (VBM region). (**a**,**b**) Cs_1–*x*_FA_*x*_PbBr_3_ on b-PEI/ZnO/ITO, (**c**,**d**) PVK on Cs_1–*x*_FA_*x*_PbBr_3_ /b-PEI/ZnO/ITO. UPS spectra of Cs_1–*x*_FA_*x*_PbBr_3_ layers were recorded in binding energy of − 2 to 12 eV for investigating the valence band electronic structures of the heterojunctions.

The effects of FA doping on the electronic structure of QD-LEDs also have been investigated. UPS measurements were carried out in the secondary electron cutoff (Fig. 5a) and/or VBM (Fig. 5b) regions for the Cs_1–*x*_FA_*x*_PbBr_3_/b-PEI/ZnO/ITO multilayer heterojunctions. From the onset values in Fig. 5a, the *Φ* (*Φ* = *hν* − |*E*_cutoff_ − *E*_Fermi_|, *hν* = 21.22 eV for He I) of each layer was calculated to be 5.06, 5.30, and 5.35 eV, for Type A, B and C, respectively. As the FA content increased from 0 to 0.055, the Φ value slightly increased from 5.06 eV to 5.35 eV. Also, we observed a slight shift of the VB level towards *E*_Fermi_ with FA doping (Fig. 5b).

**Figure 6:**
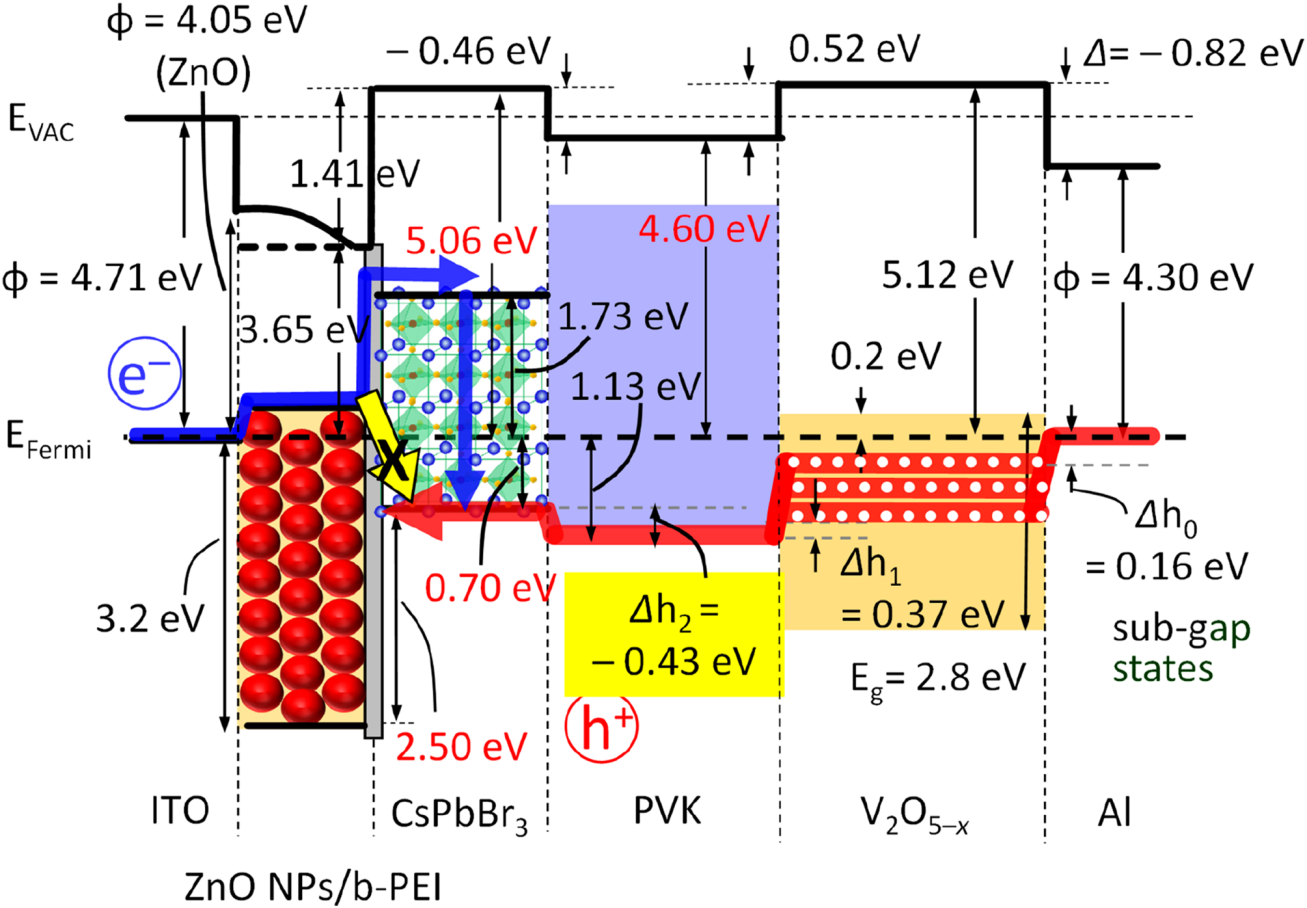
Electronic energy level alignments of CsPbBr_3_ QD-LEDs derived from UPS data.

For effective hole injection, PVK was used as a hole transfer layer (HTL). PVK layers exhibited the similar electronic energy level as Cs_1–*x*_FA_*x*_PbBr_3_. From the onset values shown in Fig. 5c, the *Φ* of PVK was calculated to be 4.60, 4.62, and 4.62 eV, for Type A, B and C, respectively. The *Φ* values of other layers were also obtained (Figure S9a, Supplementary Information). The *IE* values of PVK for all Type A, B and C were estimated as same (*IE* ~ 5.73 eV). The electronic energy level of PVK was almost unaffected by FA doped Cs_1–*x*_FA_*x*_PbBr_3_ QDs sublayer (Fig. 5c,d). The hole barrier height (Δ*h*) was estimated from the difference of HOMO (and VBM) level between overlayer and underlayer (Figures S9b,c). Due to the significantly lowered energy level which was attributed to the laying-down assembly of PVK chains on the Cs_1–*x*_FA_*x*_PbBr_3_ layer (Figure S10, Supplementary Information)^34^, PVK layer formed the quantum-well-like energy alignment rather than the hole-barrier. The Δ*h*_2_ formed at the PVK/QD heterointerface was determined to be − 0.43 eV, and which helps to inject holes to the Cs_1–*x*_FA_*x*_PbBr_3_ layer without large barrier. This was well supported by the *J*–*V* characteristics of the hole-only-device (HOD) (Figure S11a, Supplementary Information). The *J*–*V* curve of the HOD (Al/V_2_O_5–*x*_/PVK/CsPbBr_3_/ITO) was quite similar as that of HOD (Al/V_2_O_5–*x*_/PVK/ITO) while the leakage current was much lower in the low bias voltage. This imparts that there is no significant hole-barrier between CsPbBr_3_ layer and PVK layer.

b-PEI was used to block the undesirable parasitical electron injection (denoted yellow arrow in Fig. 6) due to the moderately high interface dipole at the interface of CsPbBr_3_/b-PEI and the decreased *Φ* of ZnO from 4.71 eV to 3.65 eV (1.41 eV, see Supplementary Figure S9). As shown in Supplementary Figure S11b, the excessive electron injection was well suppressed in the electron-only-device (EOD) (Al/CsPbBr_3_/b-PEI/ZnO/ITO), and which improves the charge carrier balance of Cs_1–*x*_FA_*x*_PbBr_3_ QD-LEDs.

Finally, the space-charge-limited current (SCLC) analysis was performed for EODs made of Type A, B and C to prove the reduction of trap-states by FA doping (Figure S12, Supplementary Information). The trap density (*n*_trap_) was calculated by the equation *n*_trap_ = 2εε_0_*V*_TFL_/(*ed*^2^), where *ε* is a relative dielectric constant (ε = 16.46)^42^, *ε*_0_ is the permittivity constant in free space, *e* is the elementary electronic charge, and *d* is the thickness. As shown in Supplementary Figure S12, FA doping significantly decreased the onset voltage of the trap-filled limit regime (*V*_TFL_) from 1.8 V (Type A) to 1.3 V and 1.6 V (Type B and Type C). The calculated *n*_trap_ values were 1.5 × 10^−18^ cm^−3^, 9.4 × 10^−17^ cm^−3^, and 1.2 × 10^−18^ cm^−3^ for the Type A, Type B, and Type C, respectively. This result clearly shows that the trap states were significantly reduced by FA doping.

## Conclusion

In summary, FA doped Cs_1–*x*_FA_*x*_PbBr_3_ QDs have been used for high-efficiency inverted-type QD-LEDs. Due to the capability of hydrogen bonding as well as ionic interaction with Br^‒^, FA doped in Cs_1–*x*_FA_*x*_PbBr_3_ QDs significantly increased the content of Br both at the surface and the inner part of Cs_1–*x*_FA_*x*_PbBr_3_. The compositional ratio, (Cs + FA):Pb:Br varied from 1.00:0.79:3.11 to 1.00:0.84:3.35 and 1.00:0.91:3.54 as the FA content increased from *x* = 0 to 0.04 and 0.055, respectively. The Br-enrichment in Cs_1–*x*_FA_*x*_PbBr_3_ QDs by FA doping significantly decreased the trap density (*n*_trap_), and which accordingly led to the increase of optical properties of Cs_1–*x*_FA_*x*_PbBr_3_ QDs and their QD-LEDs. PLQY of Cs_1–*x*_FA_*x*_PbBr_3_ QDs increased from 76.8% (*x* = 0) to 85.1% (*x* = 0.04), and L_*max*_ and CE_*max*_ of Cs_1–*x*_FA_*x*_PbBr_3_ QD-LEDs were improved from L_*max*_ = 2880 cd m^−2^ and CE_*max*_ = 1.98 cd A^−1^ (*x* = 0) to L_*max*_ = 5200 cd m^−2^ and CE_*max*_ = 3.87 cd A^−1^ (x = 0.04). The energy band diagram of Cs_1–*x*_FA_*x*_PbBr_3_ QD-LEDs deduced by UPS analyses revealed that the hole/electron carrier injection was well balanced because the energy barrier at CsPbBr_3_/HTL interface was significantly reduced by using PVK as a HTL while the energy barrier at ETL/CsPbBr_3_ interface was slightly increased by modifying ZnO with b-PEI.

## Methods

### Fabrication of Cs_1−*x*_FA_*x*_PbBr_3_ QDs

Colloidal perovskite CsPbBr_3_ QDs were synthesized by following the previous literature^6^. Firstly, Cs precursor solution was prepared by mixing 1 mmol CsCO_3_ (Aldrich, CAS No. 534-17-8) and 10 mL octanoic acid (OTAc; Aldrich, CAS No. 124-07-2). PbBr_2_ precursor solution was separately prepared by dissolving 1 mmol of PbBr_2_ (Aldrich, CAS No. 10031-22-8) and 2 mmol of tetraoctylammonium bromide (TOAB; Aldrich, CAS No. 14866-33-2) in 10 mL of toluene. For the synthesis of CsPbBr_3_ QDs, Cs precursor solution (1 mL) was quickly injected into PbBr_2_ precursor solution (9 mL), and then the solution was stirred for 5 min at room temperature. Afterward, didodecyldimethylammonium bromide (DDAB; Aldrich, CAS No. 3282-73-3, 3 mL) dissolved in toluene (10 mg mL^−1^) was added dropwise to the reaction solution, and which was stirred more for 5 min. Lastly, the reaction solution was rapidly quenched by cooling in ice bath. CsPbBr_3_ QDs were purified by centrifugation (g-force = 2800 RCF) to remove large particles and aggregates. After centrifugation, the colloidal CsPbBr_3_ solution (with a bright green color and a green emission) was obtained. The CsPbBr_3_ solution was further purified based on the previous reports^6^. Ethyl acetate was added to the CsPbBr_3_ solution with a 2:1 volume ratio. After centrifugation, the precipitate was collected and dispersed in toluene. The precipitate solution (in toluene) was mixed again with ethyl acetate with a 2:1 volume ratio, and which was centrifugated. Finally, the collected precipitate was re-dispersed in *n*-octane with a concentration of 10 mg/mL for further use. Formamidinium (FA) doped Cs_1−x_FA_x_PbBr_3_ QDs were also prepared by following the same procedures with precursors of FA acetate (TCI, CAS No. 3473-63-0) and CsCO_3_ together.

### QD-LED fabrication

The inverted-type green-emitting Cs_1−*x*_FA_*x*_PbBr_3_ QD-LEDs with an architecture shown in Fig. 1a were fabricated as follows: ITO-coated glass (indium tin oxide; sheet resistance of 10 Ω/sq, 180 nm thick) was cleaned by sonication in acetone, 2-propanol, and deionized water and treated with UV-ozone (15 min for each process). QD-LEDs with an active size of 2.0 × 2.0 mm^2^ were fabricated via spin-coating of zinc oxide nanocrystals^31^ (ZnO NCs; diluted in ethanol), branched polyethylenmine (b-PEI; Aldrich, CAS No. 9002-98-6, in 2-methoxyethanol), CsPbBr_3_ QDs (in *n*-octane), poly(*N*-vinylcarbazole) (PVK; Aldrich, CAS No. 25067-59-8, in chlorobenzene), and V_2_O_5–*x*_ (Aldrich, CAS No. 5588-84-1,vanadium (V) oxytriisopropoxide, diluted in isopropyl alcohol) on ITO-coated glass substrates, and which were subsequently annealed at 120, 100, 70, 160, and 120 °C for 1 min, 20 min, 5 min, 10 min, and 1 min, respectively instantly after spin-coating each layer (Fig. 1a). In this case, FA concentration in Cs_1–*x*_FA_*x*_PbBr_3_ QDs was controlled to *x* = 0 (denoted as Type A), *x* = 0.04 (denoted as Type B) and *x* = 0.055 (denoted as Type C). Aluminum (Al; 120 nm thick) cathode was deposited onto the V_2_O_5–*x*_ layer by thermal evaporation in a vacuum chamber through a patterned shadow mask, for all the QD-LEDs, electron-only devices (EODs), and hole-only devices (HODs). It is notable that all devices were processed using the spin-coating method under ambient conditions (20–24 °C and 10–30% humidity), except for the deposition of electrodes. The fabricated devices were capped with a glass lid and ultraviolet curable epoxy resin. No significant damage was observed after the coating of each layer.

### Characterizations

A bright-field high-resolution transmission electron microscopy (HR-TEM), with an acceleration voltage of 200 keV (JEOL, JEM-2100F) was used to inspect the mean diameter and crystallinity of Cs_1−*x*_FA_*x*_PbBr_3_ QDs. Energy-dispersive X-ray spectroscopy (EDX) equipped in a scanning transmission electron microscopy (STEM) (STEM-EDX) was used for elemental mapping of Cs_1−*x*_FA_*x*_PbBr_3_ QDs. X-ray diffraction (XRD) patterns were taken on an X-ray diffractometer (PANalytical, X'Pert PRO). Time-integrated photoluminescence (PL) spectra were obtained on a fluorescence spectrophotometer (PerkinElmer, LS55). The photoluminescence quantum yield (PLQY) was obtained on an absolute photoluminescence quantum yield measurement system (Jasco FP-8500) with an integrating sphere at room temperature. Optical absorbance was characterized by using an UV–Vis-NIR spectrometer (Agilent Technologies, Cary 5000). The time-resolved photoluminescence (TR-PL) were measured using a time correlated single photon counting system (Horiba Jobin Yvon iHR320). A pulsed InGaN multiple quantum-well LED (λ = 405 nm, repetition rate 1 MHz and optical pulse duration 200 ps) was used as an excitation source for the TR-PL measurements. Surface morphologies of the QDs layers were characterized by field emission scanning electron microscopy (FE-SEM, Hitachi SU-8010), and atomic force microscopy (AFM, Park Systems XE-100) with a silicon probe (Nanoworld 910 M-NCHR) under non-contact mode. The root-mean-square (rms) surface roughness was averaged from at least five different areas (5.0 × 5.0 μm^2^) of a few samples prepared in different batches. The electrical and electroluminescent properties of both types of QD-LEDs (HODs and EODs) were characterized by measuring current density–voltage–luminance (*J*–*V*–*L*) curves (Photo Research, SpectraScan PR-670; Keithley, Sourcemeter 2601). Electronic structures of the QD-LEDs, including work function (*Φ*) and energy level alignment of the heterojunction layers, were estimated by measuring the secondary electron cut-off and the valance band maximum (VBM) regions of the ultraviolet photoelectron spectroscopy (UPS, Thermo Fisher Scientific, theta probe base system). To obtain the low-energy secondary electron cut-off, a bias voltage of − 10 V was applied to the sample under normal emission geometry. The *Φ* was determined by the expression of *Φ* = *hν* − |*E*_cutoff_ − *E*_Fermi_|, where *hν* = 21.22 eV for He I. The interface/surface chemical states of the multilayer heterojunctions were characterized by the X-ray photoelectron spectroscopy (XPS, Theromo Fisher Scientific K-Alpha+, monochromatic Al Kα X-ray; *hν* = 1486.8 eV).

## Supplementary information

Supplementary file1
