# Supplementary material for: Self-defect-passivation by Br-enrichment in FA-doped Cs1−xFAxPbBr3 quantum dots: towards high-performance quantum dot light-emitting diodes
Source: Sci Rep. 2020 Sep 8;10:14758. doi: 10.1038/s41598-020-71666-8 (PMC7479606; doi:10.1038/s41598-020-71666-8)
Supplement: Supplementary file 1 — Supplementary file1 [file 41598_2020_71666_MOESM1_ESM.docx]

**Supplementary Information for**

**Self-Defect-Passivation by Br-Enrichment in FA-doped Cs_1-x_FA_x_PbBr_3_ Quantum Dots: Towards High-Performance Quantum Dot Light-Emitting Diodes**

Young Ran Park,^a^ Sangwon Eom,^b^ Hong Hee Kim,^d,e^ Won Kook Choi,^d^ and Youngjong Kang*^a,b,c^

^a^ Institute of Nano Science and Technology (INST), Hanyang University, Seongdong-gu, Seoul 04763, South Korea

^b^ Department of Chemistry, Hanyang University, Seongdong-gu, Seoul 04763, South Korea

^c^ Research Institute for Natural Sciences, Hanyang University, Seoul, 04763, Korea

^d^ Center for Opto-Electronic Materials and Devices, Korea Institute of Science and Technology (KIST), Seongbuk-gu, Seoul 02792, South Korea

^e^ Department of Materials Science and Engineering, Yonsei University, Seodaemun-gu, Seoul 03722, Korea, South Korea

*Correspondence and requests for materials should be addressed to Y.K. (E-mail: youngjkang@hanyang.ac.kr)

**This file includes:**

Supplementary Figures 1–12;

Supplementary table (S1);

Supplementary Descriptions;

Supplementary References

**Figure S1.** XRD patterns of the Cs_1–_*_x_*FA*_x_*PbBr_3_. The peaks with 2θ values of 15.1, 21.5, 26.4, 30.4, 34.2, 37.6, 43.8, 46.5 correspond to the (100), (110), (200), (210), (211), (220), and (300) planes of the CsPbBr_3_ crystal, respectively (JCPDS no. 54-0752), while the peaks of Type B and C were slightly shifted towards lower 2θ values.

**Figure S2.** FE-SEM micrographs of a) ZnO on ITO, (b) b-PEI on ZnO/ITO, (c) CsPbBr_3_ on b-PEI/ZnO/ITO, (d) PVK on CsPbBr_3_/b-PEI/ZnO/ITO, and (e) V_2_O_5–_*_x_* on PVK/CsPbBr_3_/b-PEI/ZnO/ITO.

**Figure S3.** XPS analyses of Cs_1–_*_x_*FA*_x_*PbBr_3_ QDs. (a) XPS survey spectra showing Br 3*d*, Pb 4*f*, C 1*s*, N 1*s*, O 1*s*, and Cs 3*d*. High-resolution XPS data for (b) Cs 3*d*, (c) Pb 4*f*, and (d) C 1*s*.

High-resolution C 1*s*, Cs 3*d*, and Pb 4*f* chemical states are shown in Figures S3b-d, respectively. Figure S3b (top and middle) shows the C 1*s* spectra of Cs_1–_*_x_*FA*_x_*PbBr_3_ exhibiting multiple peaks at 285 eV, which are noticeably distinguished from nearly symmetric C 1*s* peak of CsPbBr_3_ (bottom). Considering the chemical states of carbon in Cs_1–_*_x_*FA*_x_*PbBr_3_, the peak at ~285 eV can be deconvoluted into three peaks of C–C (284.8 eV), C–N (285.6 eV), and C=N (287.6 eV), respectively. Type A exhibited only two peaks of C–C and C–N without the C=N bonding states. However, a certain amount of C=N bonding at 287.6 eV was observed in Type B and C Cs_1–_*_x_*FA*_x_*PbBr_3_ which corresponds to the FA (NH_2_CH=NH_2_^+^) cation. Figure S3c shows the Cs 3*d* (3*d*_5/2_ at 724 eV and 3*d*_3/2_ at 738 eV). Similarly, Pb 4*f* exhibits 4*f*_7/2_ at 136.97 eV and 4*f*_5/2_ at 141.83eV, and which correspond to Pb^2+^ states without metallic Pb^0^ (Figure S3d).


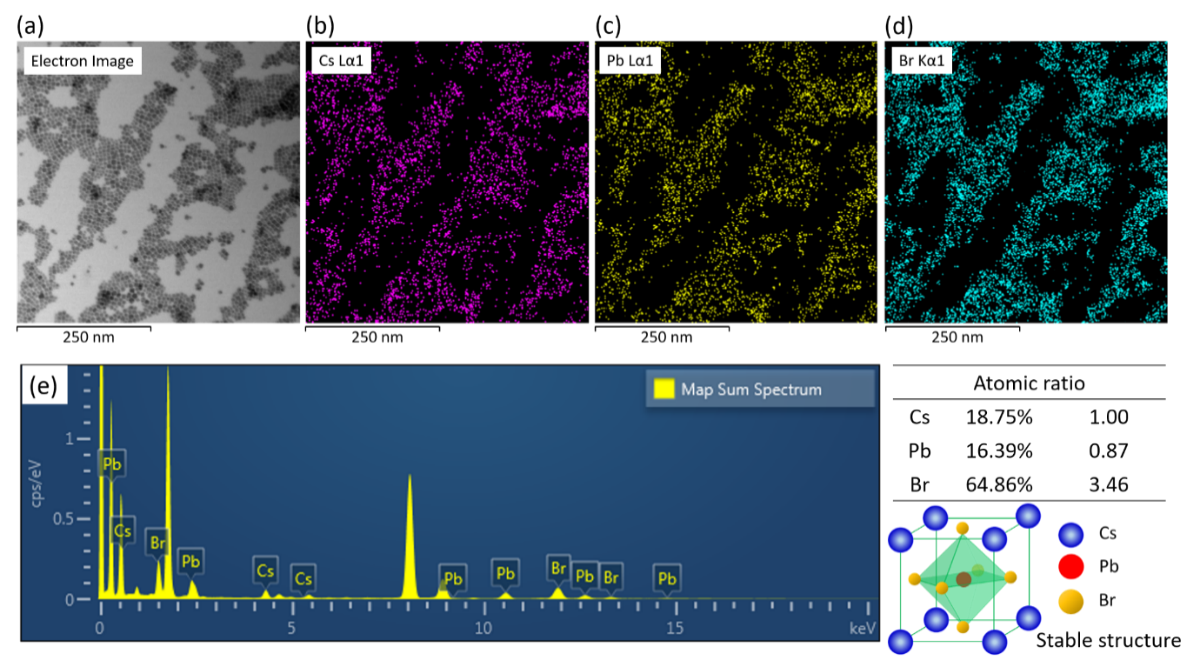


**Figure S4.** STEM-EDX mapping for undoped CsPbBr_3_ QDs. (a) A bright-field STEM micrograph of CsPbBr_3_ QDs. (b-d) The corresponding EDX mapping of CsPbBr_3_ QDs: (b) Cs (pink), (c) Pb (yellow), and (d) Br (cyan). (e) STEM-EDX spectra of CsPbBr_3_ QDs.


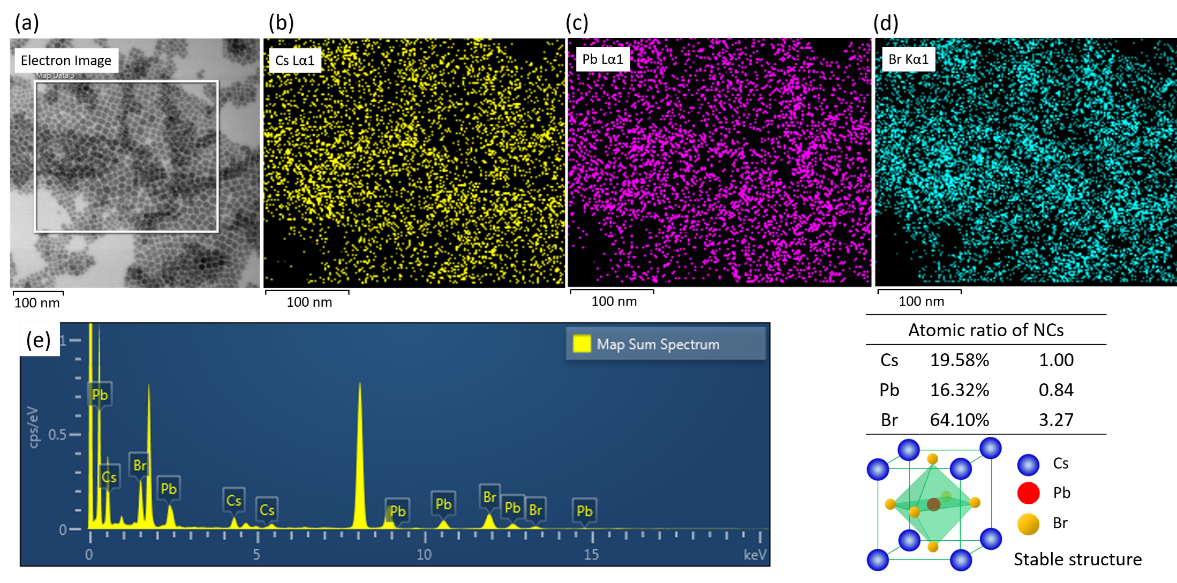


**Figure S5.** STEM-EDX mapping for undoped CsPbBr_3_ NCs. (a) A bright-field STEM micrograph of CsPbBr_3_ NCs. (b-d) The corresponding EDX mapping of CsPbBr_3_ NCs: (b) Cs (pink), (c) Pb (yellow), and (d) Br (cyan). (e) STEM-EDX spectra of CsPbBr_3_ NCs.

**Figure S6.** Absorbance (blue open circles) and emission (black solid circles) spectra of Cs_1-x_FA_x_PbBr_3_ QDs. (a) Type A, (b) Type B, and (c) Type C Cs_1-x_FA*_x_*PbBr_3_ QDs. The excitation wavelength for PL spectra was λ_exc_ = 350 nm (E_exc_ = 3.543 eV). All absorption/emission spectral pairs are offset for clarity. (d) FA content dependent absorbance and PL changes, and corresponding Stokes shift changes.[^1-3^](#_ENREF_1)

~~~~

**Figure S7.** (a) PLQY changes of Cs_1–_*_x_*FA*_x_*PbBr_3_ QD and NC solutions as a function of the storage time in air. (b) Photographs of the as-synthesized CsPbBr_3_ NC/QD crude solutions (left) and the purified Cs_1–_*_x_*FA*_x_*PbBr_3_ NC/QD solutions (right) under ambient conditions. (c) Photographs of the CsPbBr_3_ QD film under daylight (left and middle) and UV (365 nm) irradiation (right) on the multilayers of ITO/ZnO/b-PEI, respectively.

**Figure S8.** Time-resolved PL spectra for Cs_1–_*_x_*FA*_x_*PbBr_3_ QDs. TR-PL spectra of Type A (yellow circle), Type B (magenta square), and Type C (cyan triangle) measured at wavelength of 508, 512, and 513 nm, respectively. The corresponding fitted curves (black line) were derived by using iterative deconvolution fitting process based on equation, $I\left( t \right)= W_{1}e^{(-\frac{t}{\tau_{1}})}+ W_{2}e^{(-\frac{t}{\tau_{2}})}+A$, and the instrumental response function. Each amplitude *W*_i_ and decay time *τ*_i_ are summarized in Table S1.

**Table S1.** Time-resolved PL decay profiles of Cs_1–_*_x_*FA*_x_*PbBr_3_ QDs

|  | **Type A** | **Type B** | **Type C** |
| --- | --- | --- | --- |
| A | 26.31 | 28.01 | 28.53 |
| *W*_1_ (%) | 70.09 | 65.31 | 68.87 |
| *τ*_1_ (ns) | 8.47 | 8.86 | 8.96 |
| *W*_2_ (%) | 29.91 | 34.69 | 31.13 |
| *τ*_2_ (ns) | 23.37 | 39.63 | 38.56 |
| *<τ>* (ns) | 16.53 | 30.51 | 28.51 |
| *W_2_/W_1_* | 0.427 | 0.531 | 0.452 |

The PL decay curves were numerically analyzed using a bi-exponential model fitting expressed by

$I\left( t \right)= W_{1}e^{(-\frac{t}{\tau_{1}})}+ W_{2}e^{(-\frac{t}{\tau_{2}})}+A$,

where *I*, *t*, *W*, and *τ* is normalized emission intensity, time after excitation, amplitude coefficient, and decay time constant, respectively.

**Figure S9.** UPS analyses of various multilayer heterojunctions recorded in the (a) low kinetic energy region (the secondary electron cut-off), and (b) the low-binding-energy region (VBM region). (c) UPS spectra of PVK layers recorded in binding energy of −2 to 12 eV for investigating the valence band electronic structures of the heterojunctions.

In Figure S9a, the *Φ* of each layer was calculated to be 4.71, 4.05, 3.65, 5.06, 4.6, and 5.12 eV for (i) ITO, (ii) ZnO (on ITO), (iii) ZnO midified with b-PEI (on ITO), (iv) CsPbBr_3_ (on b-PEI/ZnO/ITO), (v) PVK (on CsPbBr_3_/b-PEI/ZnO/ITO), and (v) V_2_O_5–_*_x_* (on PVK/CsPbBr_3_/b-PEI/ZnO/ITO) layers, respectively. The ultrathin layer of b-PEI can significantly decrease the work function of ZnO. Also, the interface dipoles (Δ) at (ii) ZnO/ITO, (iii) b-PEI modified ZnO/ITO, (iv) CsPbBr_3_/b-PEI modified ZnO, (v) PVK/CsPbBr_3_, and (vi) V_2_O_5–_*_x_*/PVK were estimated to be 0.66, 1.06, −1.41, 0.46, and −0.52 eV, respectively, with respect to those underlayers as marked in Figure S9a. Meanwhile, the HOMO and/or VBM edges were measured at −3.26, −3.20, −0.7, −1.13, and −0.16 eV with respect to the E_Fermi_, which definitely determine the VBM (or HOMO) levels of (ii) ZnO, (iii) b-PEI modified ZnO, (iv) CsPbBr_3_, (v) PVK, and (vi) PVK layers, in equilibrium, respectively (Figure S9b). As for the electronic structures of PVK in each QD-LEDs, the UPS bands of (v) PVK on Cs_1–_*_x_*FA*_x_*PbBr_3_/b-PEI/ZnO/ITO originates from π states of the carbazole group for PVK (2.2 eV and 5.9 eV), π state of the main chain atoms (6.7 eV), σ(C–C) and σ(C–H) states distributed over the pendant group and the main chain (8.3 eV and 9.2 eV), and σ(C–H) states in the carbazole pendant group (11.1 eV) (Figure S9c), whose UPS spectra were in good agreement with those of PVK.[^4^](#_ENREF_4) This indicates that molecular orientation for PVK on Cs_1–_*_x_*FA*_x_*PbBr_3_ is the lying-down geometry because the peak of σ(C–H) (11.1 eV) was not clearly observed from PVK on QDs/b-PEI/ZnO/ITO. In addition, the PVK layer was not damaged or degraded during the device fabrication process.

**Figure S10.** Schematic illustration of interfacial chemical structure at Cs_1–_*_x_*FA*_x_*PbBr_3_ /PVK.

As schematically depicted in Figure S10, the pendent group distance of PVK is 0.85 nm[^5^](#_ENREF_5) and the diagonal distance between Br and Br on the CsBr surface is calculated at 0.83 nm for our system, therefore the pendent group distance of PVK and the distance of Br–Br atom is the similar size. There is a strong interfacial Br–H hydrogen-bonding at the QDs/PVK interface, [^6^](#_ENREF_6)^,^[^7^](#_ENREF_7) on which the benzenoid rings of PVK can be stacked with weak π–π interaction, tentatively aligned in the form of lying-down molecular geometry for the PVK molecules. Such a PVK layer on QDs was observed to have energy levels at lower energy positions and to have no Δ*h* for superior QD-LEDs performances with high hole conduction capability (Figure S11a). Accordingly, the interfacial Br-H hydrogen-bonding is a key to vary the electronic energy levels of PVK layer for designing high efficiency CsPbBr_3_ QD-LEDs.


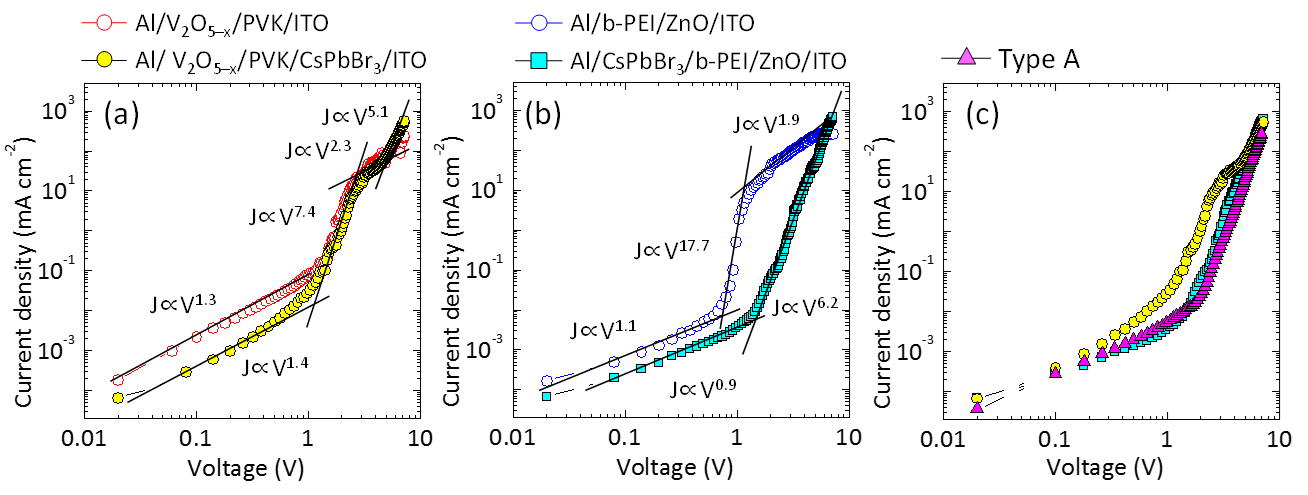


**Figure S11.** *J*–*V* characteristic curves of (a) hole-only devices (HODs), (b) electron-only device (EOD), and (c) EOD, HOD, and Type A QD-LEDs.

**Figure S12.** Estimation of trap-state density using the space-charge-limited current method, obtained using *J–V* curves of Type A (yellow circle), Type B (magenta square), and Type C (cyan triangle) EODs. The red lines represent the ohmic regime, and the blue lines indicate the trap-filled limit (TFL) regime with the onset voltage (V_TFL_).

**Supplementary References**

1 Li, X. M. *et al.* CsPbX_3_ quantum dots for lighting and displays: room-temperature synthesis, photoluminescence superiorities, underlying origins and white light-emitting diodes. *Adv. Funct. Mater.* **26**, 2435—2445 (2016).

2 Brennan, M. C. *et al.* Origin of the size-dependent stokes shift in CsPbBr_3_ perovskite nanocrystals. *J. Am. Chem. Soc.* **139**, 12201—12208 (2017).

3 Butkus, J. *et al.* The evolution of quantum confinement in CsPbBr_3_ perovskite nanocrystals. *Chem. Mat.* **29**, 3644—3652 (2017).

4 Park, Y. R. *et al.* Graphene oxide inserted poly(*N*-vinylcarbazole)/vanadium oxide hole transport heterojunctions for high-efficiency quantum-dot light-emitting diodes. *Adv. Mater. Interfaces* **4**, 1700476 (2017).

5 Tsutsui, K. *et al.* Structural chemistry of polymerizable monomers: 1. crystal-structure of *N*-vinylcarbazole. *Acta Crystallographica Section B-Structural Science* **32**, 3049—3053 (1976).

6 De Roo, J. *et al.* Highly dynamic ligand binding and light absorption coefficient of cesium lead bromide perovskite nanocrystals. *ACS Nano* **10**, 2071—2081 (2016).

7 Ravi, V. K. *et al.* Origin of the substitution mechanism for the binding of organic ligands on the surface of CsPbBr_3_ perovskite nanocubes. *J. Phys. Chem. Lett.* **8**, 4988—4994 (2017).
